# Acute Liver Rejection in a Multiple Myeloma Patient Treated with Lenalidomide

**DOI:** 10.1155/2020/8894922

**Published:** 2020-12-12

**Authors:** Iuliana Vaxman, John Eaton, Hee Eun Lee, Morie A. Gertz

**Affiliations:** ^1^Division of Hematology, Mayo Clinic, Rochester, MN, USA; ^2^Institute of Hematology, Davidoff Cancer Center, Rabin Medical Center Petah-Tikva, Israel; ^3^Sackler Faculty of Medicine Tel-Aviv University, Tel-Aviv, Israel; ^4^Division of gastroenterology and hepatology, Mayo Clinic, Rochester, MN, USA; ^5^Pathology Department, Mayo Clinic Rochester, MN, USA

## Abstract

Herein we present a patient that underwent a liver transplant due to primary biliary cholangitis (PBC) and after 9 years developed multiple myeloma. Following the cessation of mycophenolate mofetil and 2 weeks after lenalidomide treatment was started, the patient experienced acute cellular rejection. The patient recovered after treatment with corticosteroids, resumption of mycophenolate mofetil, and cessation of lenalidomide. Lenalidomide-associated allograft rejection has been reported in other organs. However, this is the first case report of liver rejection induced by lenalidomide.

## 1. Introduction

Lenalidomide has immunomodulatory, antiangiogenic, and antineoplastic characteristics and is used for the treatment of multiple myeloma (MM) [1, 2] and other hematological malignancies [3–5]. Acute cellular rejection secondary to lenalidomide has been described following kidney [6, 7] and cardiac transplantation [8–10]. This is the first case report of liver rejection induced by lenalidomide.

## 2. Case Presentation

A 65-year-old Caucasian female with a past medical history significant for infantile polio, which resulted in lower motor neuron damaged in her left leg with atrophy and weakness, was seen at Mayo Clinic. The patient was diagnosed with primary biliary cholangitis (PBC) in May 2010 and underwent a living donor liver transplant in 2011. At the first week posttransplant, she had a biopsy-proven mild acute cellular rejection and was treated with 2 doses of high dose-methylprednisolone. The patient maintained normal liver tests thereafter.

In January 2020, she presented with anemia (hemoglobin 10 g/dL). Bone marrow biopsy showed 65% plasma cells, lambda light chain restricted, and fluorescence in situ hybridization (FISH) indicated a plasma cell clone with a 1q duplication, monosomy 13, and monosomy 14. In addition, a tetraploid subclone was observed in 20% of the nuclei. Epstein-barr virus hybridization was not performed so it is uncertain whether myeloma is a variant of posttransplant lymphoproliferative disorder (PTLD). The diagnostic workup included WBC 2.9 × 10^9^/L (*N* = 3.4‐9.6), platelet count 128 × 10^9^/L (*N* = 157‐371), calcium 9.9 mg/dL (*N* = 8.8‐10.2), creatinine 0.73 mg/dL (*N* = 0.59‐1.04), albumin 3.8 g/dL (*N* = 3.5‐5), and beta 2 microglobulin 3.5 *μ*g/mL (*N* = 1.2‐2.7). M spike 0.1 g/dL type lambda, IgG 982 mg/dL (*N* = 767‐1590), IgA 33 mg/dL (*N* = 61‐356), and IgM 47 mg/dL (*N* = 37‐286). Serum lambda 290 mg/dL (*N* = 0.57‐2.63), serum kappa 1.23 mg/dL (*N* = 0.33‐1.94), and kappa to lambda ratio was 0.003 (*N* = 0.26‐1.65). Her 24-hour urine protein was 50 mg, and no monoclonal protein was seen. CT skeletal survey was negative for lytic lesions. Liver tests were normal. Multiple myeloma (MM) was diagnosed. At MM diagnosis, AST was 31 U/L (*N* = 8‐43), ALT was 23 (*N* = 7‐45), ALP was 69 U/L (*N* = 35‐104), and bilirubin was 0.47 (*N* = 0‐1.2).

Immunosuppression following liver transplant consisted of mycophenolate mofetil 500 mg twice daily and tacrolimus 0.5 mg twice daily. When MM was diagnosed, mycophenolate mofetil was stopped due to cytopenias and the need for myeloma directed therapy. The patient started bortezomib 1.3 mg/m^2^ once weekly, lenalidomide 25 mg 21/28 days, and dexamethasone 40 mg once weekly (VRd). After the first 2 weeks of therapy, in cycle 1, she had a rise in AST to 365 U/L (*N* = 8‐43), ALT to 386 (*N* = 7‐45), and ALP to 253 U/L (*N* = 35‐104). Bilirubin was elevated to at 3.3 mg/dL (*N* = 0‐1.2) and 1.4 mg/dL direct. VRd treatment was discontinued. Preceding the rise in liver tests, her tacrolimus trough was 4.0 ng/mL.

A liver biopsy was performed and revealed the following findings: (i) mild to moderate portal inflammation with plasma cells and eosinophils and necroinflammatory foci in the lobular parenchyma, (ii) focal interface activity, (iii) endotheliitis in the portal tracts and hepatic vein branches, (iv) focal cholangitis with biliary epithelial injury and granulomatous inflammation, and (v) trichome stain was negative for fibrosis. This was felt to represent recurrent PBC with mild acute cellular rejection. In addition, the prominent interface and lobular inflammatory activity raised the possibility of a minor component of superimposed drug-induced liver injury or concurrent autoimmune hepatitis.

**Figure 1:**
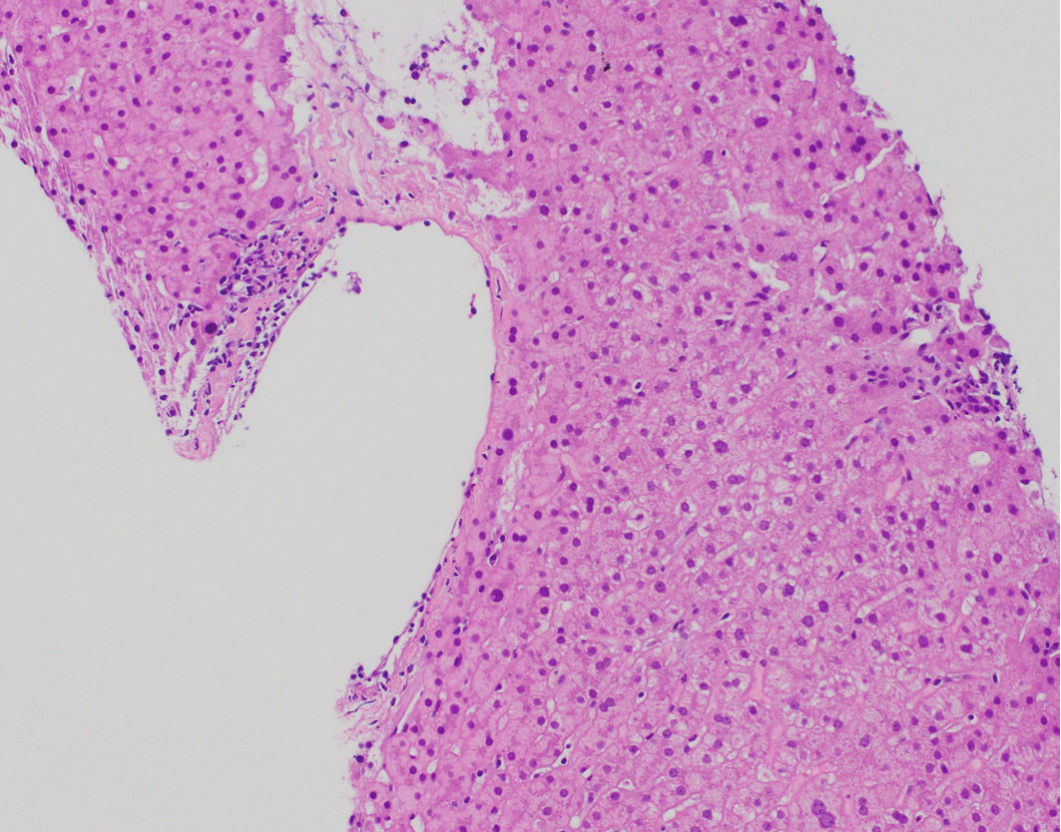
The liver biopsy shows venous endotheliitis which is one of the diagnostic features of acute cellular rejection (hematoxylin and eosin stain, original magnification ×200).

She was started on 1000 mg IV methylprednisolone, mycophenolate mofetil was resumed at 250 mg twice daily, and tacrolimus was raised to 1 mg twice daily. Treatment with ursodeoxycholic acid 15 mg/kg/day was started. A second biopsy was done a week after the first one which showed improving but persistent features of acute cellular rejection. No significant interface or lobular inflammatory activity was seen on the biopsy which may be due to the prior methyl prednisone treatment. Features of recurrent PBC were not visualized on this biopsy which may be related to the patchy nature of PBC (Figure 1).

The patient received a total of 3 doses of methylprednisolone 1000 mg (days 1, 3, and 5). Liver enzyme was normalized within a month, and MM treatment was resumed which included bortezomib 1.3 mg/m^2^ once weekly, cyclophosphamide 500 mg once weekly, and dexamethasone (VCd). Pre bone marrow transplant, the patient achieved a very good partial response (VGPR) as evidenced by serum lambda light chain reduction of more than 90%. Liver enzymes remain normal.

## 3. Discussion

We present a patient that underwent a liver transplant due to PBC and after 9 years developed MM. Following the cessation of mycophenolate mofetil and 2 weeks after lenalidomide treatment was started, the patient experienced acute cellular rejection and ultimately recovered after treatment with corticosteroids, resumption of mycophenolate mofetil, and cessation of lenalidomide. While the cessation of mycophenolate mofetil in this case may have been a contributing factor, lenalidomide-associated allograft rejection has been reported in other organs and is important for clinicians to be aware of.

While acute cellular rejection secondary to lenalidomide and other immunomodulatory treatments (IMiDs) has not previously been reported after liver transplant, it has been described following kidney [6, 7] and cardiac transplantation [8–10]. Hence, the possibility of IMiD-induced rejection may not be organ specific. Indeed, a heart rejection 40 days after pomalidomide initiation was reported [9]. In all case reports, organ rejection occurred early in the courses of lenalidomide treatment, always in the first 2 months from IMiD treatment initiation. In most of the reported cases, organ function improved after antirejection treatment.

Lenalidomide is used in the treatment of MM [1], light chain amyloidosis [5], mantle cell lymphoma [11], deletion 5q myelodysplastic syndrome [4], diffuse large B cell lymphoma [12], and chronic lymphocytic leukemia [3].

The underlying mechanism leading to rejection is unclear. IMiDs activate the cereblon complex which degrades IKZF3, a repressor of interleukin 2 (IL-2). This causes an increase in IL-2, leading to the recruitment of CD4+ cells and natural killer cells, which may cause an immune rejection of the solid organ. IMiDs also induce T cell activation by stimulating CD28, which works via nuclear factor kappa B [13].

It is interesting to note that there are reports about lenalidomide causing graft versus host disease (GVHD) after autologous [14] and allogeneic [15] stem cell transplantations. In a prospective trial, 9 out of 24 patients treated with lenalidomide after allogeneic transplantation developed GVHD, and the median time was 22 days. An increased risk of autoimmune disease in MM patients treated with IMiDs was reported in a retrospective trial, most cases early after treatment initiation [16].

## 4. Conclusion

Transplantation physicians and hematologists should be aware of the potential risk of organ rejection associated with immunomodulatory drugs such as lenalidomide. Patients who are receiving IMiDs (thalidomide, lenalidomide, or pomalidomide) who have previously undergone a transplant should have their allograft function monitored closely.

## Data Availability

This is a case report. References from PUBMED are included in the manuscript.
